# Expression profiling of mRNA and functional network analyses of genes regulated by human papilloma virus E6 and E7 proteins in HaCaT cells

**DOI:** 10.3389/fmicb.2022.979087

**Published:** 2022-09-14

**Authors:** Renjinming Dai, Ran Tao, Xiu Li, Tingting Shang, Shixian Zhao, Qingling Ren

**Affiliations:** ^1^The Affiliated Hospital of Nanjing University of Chinese Medicine, Nanjing, China; ^2^Laboratory of Clinical Applied Anatomy, Department of Human Anatomy, School of Basic Medical Sciences, Fujian Medical University, Fuzhou, China

**Keywords:** cell adhesion, cervical epithelioma, differentially expressed genes, HPV, oncogenes, RNA sequencing

## Abstract

Human papillomavirus (HPV) oncogenes E6 and E7 are essential for HPV-related cancer development. Here, we developed a cell line model using lentiviruses for transfection of the HPV16 oncogenes E6 and E7 and investigated the differences in mRNA expression during cell adhesion and chemokine secretion. Subsequently, RNA sequencing (RNA-seq) analysis was performed to explore the differences in mRNA expression. Compared to levels in the control group, 2,905 differentially expressed mRNAs (1,261 downregulated and 1,644 upregulated) were identified in the HaCaT-HPV16E6E7 cell line. To predict the functions of these differentially expressed genes (DEGs) the Gene Ontology and Kyoto Encyclopedia of Genes and Genomes databases were used. Protein–protein interactions were established, and the hub gene was identified based on this network. Real-time quantitative-PCR (RT-qPCR) was conducted to confirm the levels of 14 hub genes, which were consistent with the RNA-seq data. According to this, we found that these DEGs participate in the extracellular matrix (ECM), cell adhesion, immune control, and cancer-related signaling pathways. Currently, an increasing number of clinicians depend on E6/E7mRNA results to make a comprehensive judgment of cervical precancerous lesions. In this study, 14 hub genes closely related to the expression of cell adhesion ability and chemokines were analyzed in HPV16E6E7-stably expressing cell lines, which will open up new research ideas for targeting E6E7 in the treatment of HPV-related cancers.

## Introduction

The human papillomavirus (HPV) is a DNA virus that encodes approximately eight genes. It can cause cancers of the antrum, genital tract, upper respiratory tract, and digestive tract (Dunne and Park, 2013). High-risk HPV E6 and E7 proteins reduce immune recognition by disrupting cell adhesion, polarity, and transcription via several pathways, such as the cell adhesion pathway (Oldak et al., 2006; Whiteside et al., 2008; Howie et al., 2009; Läubli and Borsig, 2019; Kombe Kombe et al., 2020). Studies have shown that the cervical squamous epithelium, which is the target of HPV, consists of keratinocytes, immature dendritic cells (DCs), and Langerhans cells (LCs), all of which perform immunosurveillance functions on the epithelium (Schiffman et al., 2007). LCs are the only specialized antigen-presenting cells found in the epidermis, and their abnormal expression affects antiviral immune responses. HPV-associated cervical squamous epithelial neoplasia (SIL) is strongly associated with immature LCs (Habbous et al., 2012). Cell adhesion between cells and the extracellular matrix (ECM) is required for the integrity of the epidermis and the formation of an immune barrier (Honig and Shapiro, 2020). Cell surface adhesion molecules, the major components of cell-to-cell and cell-to-extracellular interactions (Login et al., 2019), are frequently expressed in epithelial (Sumigray and Lechler, 2015), endothelial (Schimmel and Gordon, 2018), and immune (Dustin, 2009) cells. They consist of intracellular and extracellular components (CC) and can be divided into four types, cadherins, integrins, selectins, and immunoglobulins. Many studies have focused on malignant metastasis and cell adhesion (Janiszewska et al., 2020); however, the mechanisms related to interactions among HPV proteins, cell adhesion, and the epithelial immune response remains unclear. Activated leukocyte cell adhesion molecule (ALCAM) (Ferragut et al., 2021) is a cell-surface glycoprotein that has been recognized as a vital prognostic marker for various tumors, including HPV-associated head and neck squamous cell carcinoma (Zhu et al., 2020). Based on current research, ALCAM can regulate adhesion to immune cells (Te Riet et al., 2014) and control disease progression by affecting cell proliferation, migration, and invasion (von Lersner et al., 2019).

Chemokines are the largest subfamily of cytokines (Bule et al., 2021). It is well known that HPV is capable of preventing a robust immune response to the infected cells based on many mechanisms, such as by reducing the expression of chemokines (Attademo et al., 2020; Acevedo-Sánchez et al., 2021). When chemokines are unable to guide immune cells to migrate to the infected site in an orderly fashion, immune tolerance collapses and immune monitoring becomes ineffective (Kolaczkowska and Kubes, 2013).

With the increasing prevalence of Illumina technology in recent years, RNA-sequencing (RNA-Seq) technology has been effectively used in biomedical research (Tao et al., 2021; Wang et al., 2021). The carcinogenetic effect of E6 and E7 is apparent, but molecules that are induced by these proteins in HaCaT cells still need to be researched, and high-throughput sequencing would contribute vastly to this question. In this research, the differential mRNA expression profiles of HPV16E6E7-stably expressing cell lines were determined via RNA-seq. Our results showed that transcriptome levels were significantly different between the two groups of non-transfected control and HPV16E6E7-stably expressing cell lines. Transcriptome data are essential in providing a research target for mechanistic studies aimed at reversing cervical epithelioma and blocking the malignant progression of epithelial cells.

## Materials and methods

### Cell adhesion assays

E6-IREs-E7 was constructed and inserted into the plenti-Gm-cMV vector to obtain the vector plasmidpLenti-III-HPV-16E6-IRES-E7Vector (Puro). It was co-transfected with Lenti-ComboPackingMix and lentifectin into HEK293T. Cell supernatants were collected after the transfection, and the lentivirus was designated as Lenti-III-HPV-16E6-IRES-E7Virus (Puro). HaCaT cells were seeded onto 6-well plates at a density of approximately 20–30%, and then were infected with the lentivirus and incubated at 5% CO2, 37°C for 48 h. The infected HaCaT cells were plated in 96-well plates forstable transfection strain resistance screening. After HaCaT in logarithmic growth phase was infected by Lenti-III-HPV-16 E6-IRES-E7 Virus (Puro), four monoclonal cell lines, clone#4, clone#6, clone#10, clone#11, were obtained. Clone#11 showed the highest expression of target gene, validated by Real-time quantitative-PCR (RT-qPCR). Our team collaborated with ABM (Applied Biological Materials, Jiangsu, China) to establish this HaCaT-HPV16E6/E7. According to the introduction of the commercial cell adhesion kit (BestBio, Shanghai, China), the adhesion ability of parental HaCaT and HaCaT-HPV16E6E7 cells was defined. The optical density was recorded at 450 nm using a microplate reader (TECAN, SCHWEIZ). Finally, the level of cell adhesion was analyzed based on the OD value.

### Western blot analysis

Protein samples were extracted from parental HaCaT and HaCaT-HPV16E6/E7 Clone#11 cell which were harvested in three different generations using RIPA buffer (Beyotime Biotechnology, Shanghai, China). Determination of total protein was performed using a commercial kit (Beyotime Biotechnology, Shanghai, China), and then, samples were incubated at 100°C for 10 min. Protein samples were separated with 2–8% precast gels (ACE Biotechnology, Nangjing, China) and transferred onto PVDF (Millipore, Massachusetts, United States). The membranes were then soaked with 5% skim milk at 20–22°C for 1 h. After this, the solution was removed, and the membranes were incubated with primary antibodies, including those against ALCAM (1:1,000, Abmart, T57159), HPV16 E7 (1:500, bs-4623R), and HPV16 E6 (1:500, Abmart, V028329). The following day, the membrane was incubated with the secondary antibody (1:5,000, Cell Signaling Technology, 7076/7074) at 20–22°C for 1 h. The protein bands were developed using an ultrasensitive luminescent liquid reagent (Tanon, Shanghai, China) and analyzed using Image Lab version 5.1. The band density of the target protein to that of β-actin was used as the relative target protein content. All western blot experiments consisted of at least 3 independent replicates.

### Real-time quantitative-PCR

We examined expression levels of the chemokine genes *CCL2*, *CCL3*, *CCL5*, *CCL7*, and *CXCL10* using RT-qPCR. The mRNA levels were determined by real time PCR. Total RNA was extracted from the parental HaCaT and HaCaT-HPV16E6/E7 Clone#11 cell which were harvested in three different generations using an RNA extraction kit (Vazyme, Nanjing, China). The cDNA product was obtained via reverse transcription using a commercial regent at 37°C for 15 min and then 85°C for 5 s (Vazyme, Nanjing, China). GAPDH and β-actin were used as internal controls. Primers were pre-designed and synthesized from a company (Sangon Biotech, Shanghai, China), and primer sequences are described in Table 1. Products were amplified using QUANTSTUDIO 7 FLEX QUANTSTUDIO (ABAppliedBiosystems, United States) according to the instructions for the Taq Pro Universal SYBR qPCR Master Mix kit (Vazyme, Nanjing, China). RT-qPCR was performed in technical triplicate for each biological replicate. Finally, Real-time PCR results were calculated according to the ΔΔCT method by using GAPDH and β-ACTIN as housekeeping genes.

**TABLE 1:** Primer lists.

Gene	Forward primer (5′–3′)	Reverse primer (5′–3′)
GAPDH	CATCATCCCTGCCTCTACTG	CTGCTTCACCACCTTCTTG
β-actin	GGCACCCAGCACAATGAAG	CCGATCCACACGGAGTACTTG
CCL2	CAGCCAGATGCAATCAATGCC	TGGAATCCTGAACCCACTTCT
CCL5	CCAGCAGTCGTCTTTGTCAC	CTCTGGGTTGGCACACACTT
CCL7	GCCTCTGCAGCACTTCTGTG	CACTTCTTGTGTGGGGTCAGC
CXCL10	GTGGCATTCAAGGAGTACCTC	TGATGGCCTTCGATTCTGGATT
CCL3	CAGCCAGATGCAATCAATGCC	TGGAATCCTGAACCCACTTCT
CDH1	CGAGAGCTACACGTTCACGG	GGGTGTCGAGGGAAAAATAGG
CDH2	TCAGGCGTCTGTAGAGGCTT	ATGCACATCCTTCGATAAGACTG
EGF	TGGATGTGCTTGATAAGCGG	ACCATGTCCTTTCCAGTGTGT
FGF2	AGAAGAGCGACCCTCACATCA	CGGTTAGCACACACTCCTTTG
BDNF	GGCTTGACATCATTGGCTGAC	CATTGGGCCGAACTTTCTGGT
SOX2	GCCGAGTGGAAACTTTTGTCG	GGCAGCGTGTACTTATCCTTCT
TLR4	AGACCTGTCCCTGAACCCTAT	CGATGGACTTCTAAACCAGCCA
IGF1	GCTCTTCAGTTCGTGTGTGGA	GCCTCCTTAGATCACAGCTCC
DLG4	TCGGTGACGACCCATCCAT	GCACGTCCACTTCATTTACAAAC
COL1A1	GAGGGCCAAGACGAAGACATC	CAGATCACGTCATCGCACAAC
FYN	ATGGGCTGTGTGCAATGTAAG	GAAGCTGGGGTAGTGCTGAG
CXCR4	ACTACACCGAGGAAATGGGCT	CCCACAATGCCAGTTAAGAAGA
WNT5A	ATTCTTGGTGGTCGCTAGGTA	CGCCTTCTCCGATGTACTGC
IL-1β	TCGCCAGTGAAATGATGGCT	TGGAAGGAGCACTTCATCTGTT

### RNA-Seq and gene expression analysis

RNA samples harvested from the parental HaCaT and HaCaT-HPV16E6/E7 Clone#11 cell, which were harvested in four different generations and were used for sequencing. Libraries for RNA-Seq were obtained from total RNA, and sequencing was performed on the NovaSeq 6000 platform by Gene *Denovo* Biotechnology Co., Ltd (Guangzhou, China). To quantify gene expression, HTSeq was used to obtain the original read count for each gene; alternatively, reads per kilobase of transcript per million mapped reads (RPKM) were used depending on the total map read count and the gene length for each sample. DEGs were determined based on the gene expression level measured using log_2_-transformed RPKM (| log_2_FC| (fold change) > 2, FDR < 0.01). The read counts data were entered and analyzed using EdgeR and DESeq2 software, including normalization of read counts and calculation of *P*-values and FDR values. Transcriptome data (SRA accession: PRJNA850539) were stored in the NCBI SRA database.^1^

### Enrichment analysis

GO enrichment analysis includes biological process (BP), molecular function (MF), and CC, three aspects that are based on significantly enriched DEGs. The differential genes are mapped to each term of the GO database and the number of genes per term is counted to obtain a list of genes with a certain GO function and gene number statistics. Hypergeometric tests were applied to identify GO terms that were significantly enriched in differential genes compared to the whole genomic background. Briefly, all DEGs were mapped to GO terms in the GO database.^2^ Then, gene numbers were calculated for every term, and GO terms associated with significantly enriched DEGs compared to the genome background were defined using a hypergeometric test. The calculated *p*-value was subjected to false discovery rate (FDR) correction, with FDR ≤ 0.05 as the threshold. Pathways meeting this condition were defined as significantly enriched pathways among the DEGs. The KEGG analysis was carried out according to KEGG.^3^ GSEA was also performed with the GSEA software.^4^

### Generation of the protein–protein interaction network

The protein–protein interaction (PPI) network was constructed using the String database,^5^ for which genes and interactions were determined and designated as nodes and lines, respectively, in the network. To show the biological interactions among DEGs, their relationship network was visualized using Cytoscape (v3.9.2) software. Moreover, the cytoHubba plug-in was used to mine the hub genes using the MNC, Stress, Degree, Closeness, and Radiality calculation method in the Cytoscape MCODE plug-in Cytoscape, which was used to further extract the core sub-networks.

### Statistical analysis

Data are presented from three independent experiments based on the means ± SEMs. For statistical analyses, GraphPad Prism 9 software was adopted (GraphPad, United States). When comparing two groups, unpaired *t*-tests were used, and differences among groups were analyzed using one-way ANOVA. A *p*-value < 0.05 was considered statistically significant.

## Results

### Human papillomavirus 16 oncogenes E6 and E7 affect cell adhesion in HaCaT cells

**FIGURE 1:**
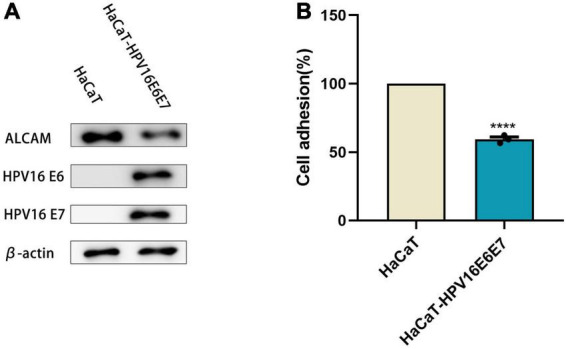
E6 and E7 injured the ability of cell adhesion in HaCaT **(A)** WB results showed that the expression of β-actin as an internal reference HPV16 E6E7, ALCAM was reduced in protein level. **(B)** The OD value of HaCaT-HPV16E6E7 was found to be significantly reduced in comparison with HaCaTHPV16 oncogenes E6 and E7 affect the secretion of chemokines in HaCaT. ****p < 0.0001.

HaCaT cells stably expressed the E6 and E7 proteins after lentiviral transduction (Supplementary Figure 1). A cell adhesion reagent kit was used to determine whether this inhibited cell adhesion by measuring the optical density value in each group of cells. The overexpression of E6 and E7 significantly reduced the cell absorbance, indicating that they negatively affected cell adhesion (Figure 1B). Simultaneously, the protein expression level of the cell adhesion molecule (CAM) ALCAM was significantly decreased when examined via western blotting analysis (Figure 1A and Supplementary Figures 2, 3).

### Human papillomavirus16 oncogenes E6 and E7 affect HaCaT cell adhesion

**FIGURE 2:**
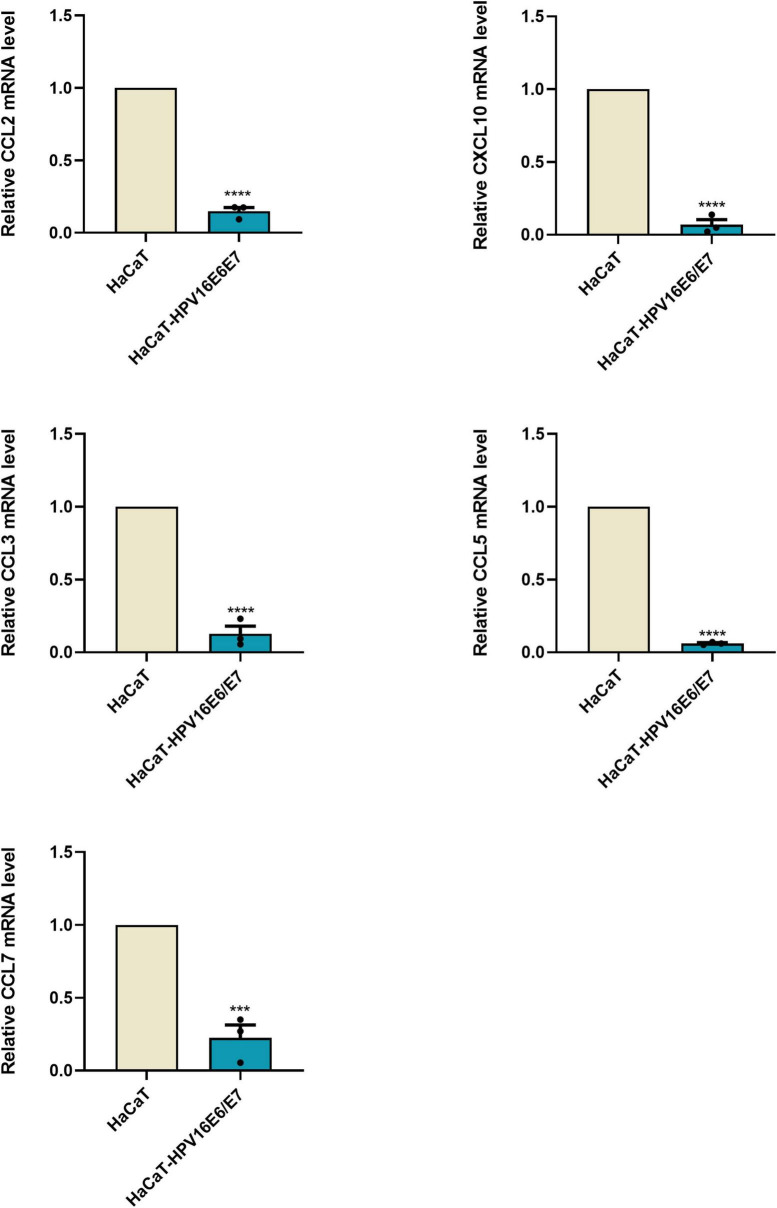
E6 and E7 damaged the secretion of chemokines in HaCaT RT-qPCR results were quantified by real-time PCR using GAPDH and β-ACTIN as internal control genes and presented in bar graph. It can be seen that overexpression of E6E7 does lead to a decrease in the relative expression of CCL2, CCL3, CCL5, CCL7, and CXCL10. ***p < 0.001, ****p < 0.0001.

Chemokines are effective chemical attractants for natural killer cells, macrophages, and monocytes, and they engage in the host immune response against HPV-infected cervical epithelia. CCL2, CCL3, CCL5, and CCL7 attract monocytes/macrophages to exert anti-infective, anti-tumor, and immunomodulatory effects at sites of inflammation. In response to pro-inflammatory stimuli, such as IL-1, TNF-α, LPS, or viruses, the expression of CCL2, CCL3, CCL5, and CXCL10 recruit immune cells to sites of inflammation. However, after overexpression of the HPVE6E7 protein, the expression levels of CCL2, CCL3, CCL5, CCL7, and CXCL10 decreased with different magnitudes. The selective loss of chemokine expression, including that of CCL2, was observed after lentiviral transduction. Moreover, their mRNA levels were significantly lower than those in keratinocyte HaCaT cells (Figure 2 and Supplementary Figure 4).

### Overview of differential mRNA expression

**FIGURE 3:**
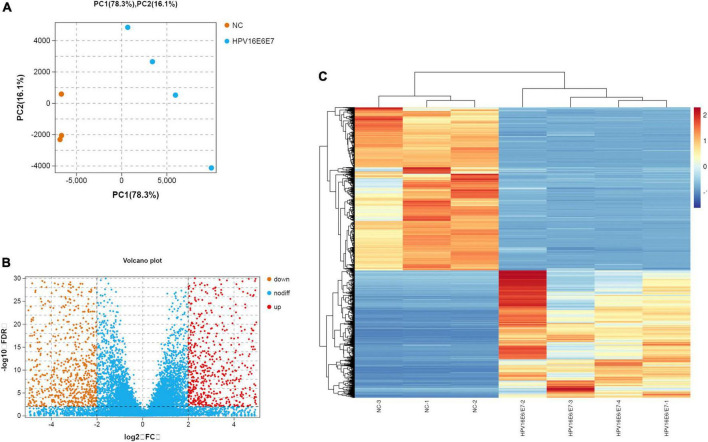
Overview of the differential mRNA expression between two groups **(A)** Use R software to carry out PCA, the horizontal and vertical coordinate represent PC1 and PC2 representatively; The distance in the graph reflects the degree of the difference between groups. **(B)** In the volcano plot, orange and red represent down- and up-regulated genes, respectively, while blue expresses genes that are not significantly different. **(C)** In the Heat Map, the closer the value of the vertical coordinate to red indicates a greater relative gene expression, and vice versa.

The original RNA-seq data (Read Count) and differential expression analysis results (DESeq2_analysis_results) used in this study were uploaded, and these tables can be found in the Supplementary Material 1. To identify DEGs between the cell groups, the Limma package was used to analyze data. The criteria were as follows: | log_2_FC| > 2 and *p* < 0.01 (Figure 3A). After this step, the mRNA expression of 1,644 genes were found to be upregulated, whereas that of 1,261 genes was downregulated; according to our data, DEGs based on mRNA expression were presented as a volcano map using the ggplot2 package (Figure 3B). Heatmaps of the DEGs were created with the heatmap package (Figure 3C).

### Enrichment analyses of differentially expressed mRNAs

**FIGURE 4:**
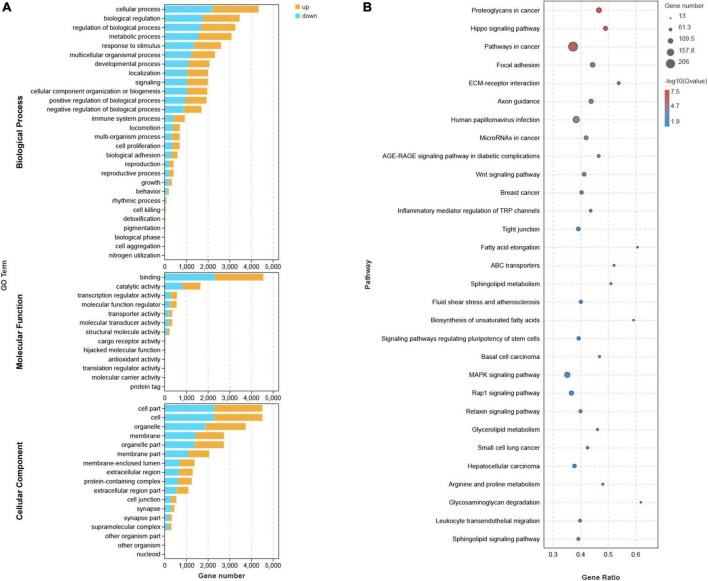
GO, KEGG Analysis. **(A)** Each bar in GO analysis represents a pathway, where the up-regulated and down-regulated genes involved in this pathway are indicated in yellow and blue, respectively. **(B)** In KEGG Pathway analysis, the size of the circle indicates the number of enriched genes.

The top 20 KEGG pathways (Figure 4A) indicated that DEGs were enriched in the Hippo and Wnt pathways and signaling pathways that regulate the ECM receptor interaction, among others. These signaling pathways exhibit crosstalk with cell adhesion by regulating the transcription of chemokines, CAMs, and growth factors, including cadherin 1 (CDH1), CDH2, epidermal growth factor (EGF), brain-derived neurotrophic factor (BDNF), interleukin (IL)-1β, fibroblast growth factor (FGF2), disks large homolog 4 (DLG4), Toll-like receptor 4 (TLR4), insulin-like growth factor 1 (IGF1), collagen type I alpha 1 (COL1A1), CXCR4, and WNT5A. In the GO analysis, DEGs closely related to several BP, MF, and CC included genes involved in adhesion, immune system, cell aggregation, transcription regulator activity, and cell junction (Figure 4B). In addition, GESA and KEGG analyses showed similar results, such as genes involved in tight junctions (Supplementary Figure 5).

### Analysis of protein–protein interaction network

**FIGURE 5:**
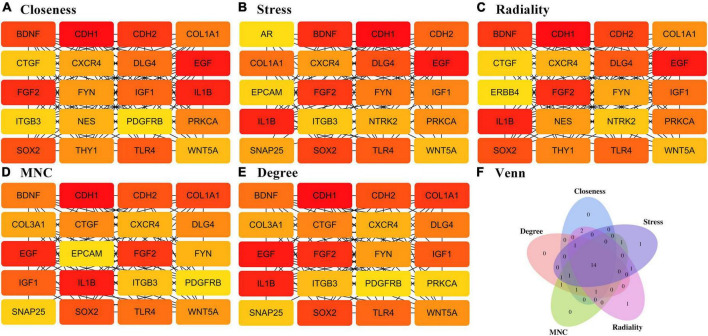
PPI network based on DEGs. **(A–E)** Different algorithms for interaction analysis of the top 20 pivotal genes. **(F)** Wayne diagram showing the 14-hub gene intersected by different algorithms.

**FIGURE 6:**
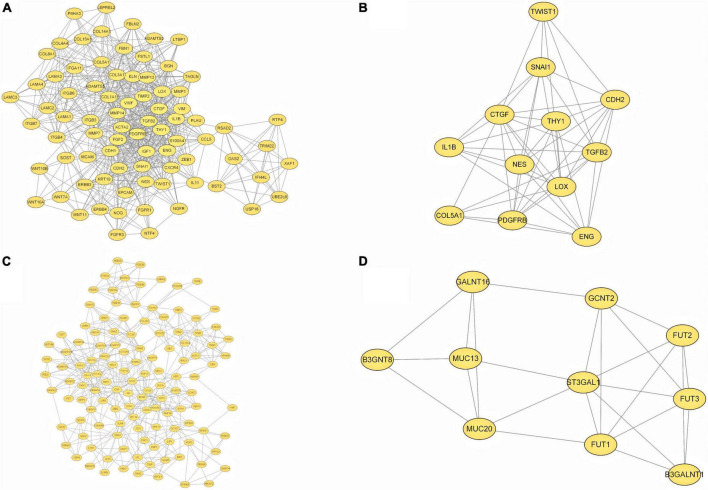
PPI network based on DEGs. **(A–D)** The top four interactive density region in the PPI network by “MCODE” plug-in was also discovered.

To provide a better understanding of interactions among the DEGs, their corresponding PPI network was generated using Cytoscape software and the STRING database. The “cytoHubba” plug-in identified the 14 core genes based on differences in the level of linkage (Figures 5A–E). These 14hub genes are obtained by taking the intersection of the DEGs obtained from five different algorithms, and are presented with the form of Venn diagram (Figure 5F). The interactive density region in the PPI network was also determined using the “MCODE” plug-in (Figures 6A–D). The figure below shows the densest regions (Figure 5E). For example, BDNF binds to CDH2 and CXCR4, TLR4 to CXCR4 and IL-1β, and CDH1 to FYN and IGF1. In this study, it was considered that these proteins play useful roles in regulating chemokine binding and macrophage-mediated cytokine production.

### Verification of differentially expressed mRNAs

**FIGURE 7:**
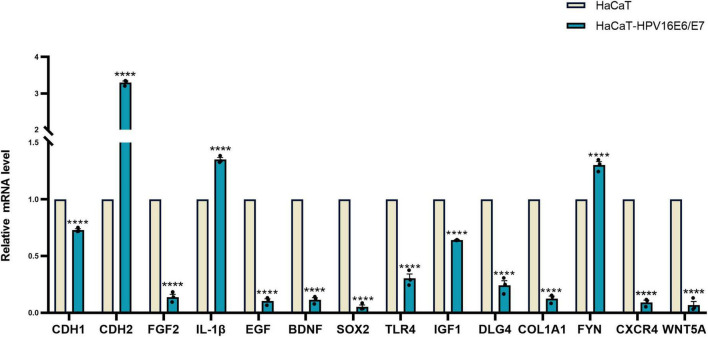
Verification of 14 hub gene Relative expression results of 14 hub gene were quantified by real-time PCR using GAPDH and β-ACTIN as internal control genes and shown by the bar graph whose horizontal and vertical coordinates represent the gene names and relative expressions, respectively. The verification is consistent with High-throughput sequencing results. ****p < 0.0001.

RT-qPCR was performed to verify the transcriptome sequencing results before and after lentiviral transduction and define the possible signaling network associated with the E6 and E7 oncogenes upon HPV16 infection. The RT-qPCR data showed that the expression of *CXCR4* was decreased in cells overexpressing the HPV16 oncogenes E6 and E7, as with the findings of RNA-seq. However, *SOX2* and *FYN* expression levels were overexpressed, in contrast to RNA-seq results. The mRNA expression levels of *CDH1*, *CDH2*, *EGF*, *IL-1*β, *FGF2*, *BDNF*, *TLR4*, *IGF1*, *DLG4*, *COL1A1*, *CXCR4*, and *WNT5A* were analyzed by RT-qPCR, and their expression was in agreement with the outcomes of RNA-Seq. (Figure 7 and Supplementary Figure 6).

## Discussion

It is well known that E6 and E7 can attach to the ECM of the cervical squamous epithelium. Further, HPV E6 and E7 can be detected in a substantial proportion of women with cervical intraepithelial neoplasia (CIN) (Guan et al., 2012); they also serve as attractive targets for HPV-related diseases (Hoppe-Seyler et al., 2018). Owing to its specific sites and functions in antigen presentation, LCs are considered essential for the immune response against HPV infection (Cunningham et al., 2010). In the epidermis of mice (Kaplan et al., 2005) and female genital tract (Britto et al., 2020), LCs produce effector cytokines to stimulate T lymphocytes and B lymphocytes through interactions with C-type lectin (CLR receptors) and TLRs. Hubert et al. (Hubert et al., 2005) confirmed decreased levels of *CDH1* mRNA and numbers of CD1α^+^ LCs in squamous cell carcinoma and SILs compared with those in normal cervical epithelia. According to the high throughput screening of clinical specimens (Britto et al., 2020), the decrease observed in the mRNA levels of *CDH1*, *Claudin 1*, *Claudin 4*, and *ZO-1* mRNA and IFN signaling upon HPV infection indicate that it could result in weakness of the epithelial barrier (Aggarwal et al., 2019; Giorgi Rossi et al., 2021). Collectively, these findings suggest that the E6 and E7 oncoproteins are strongly associated with cell adhesion in HPV-induced immunosuppression. The loss of cell adhesion caused by the imbalance in adhesion molecules and chemokines might be another immune-escape strategy that has evolved in HPV.

CXC motif chemokine ligand 10 (CXCL10), CC motif chemokine ligand 2 (CCL2), CCL3, and CCL5 are pro-inflammatory chemokines that actively participate in the inflammatory response after viral exposure (Kolaczkowska and Kubes, 2013). CCL2 (Nakamura et al., 1995; Kunstfeld et al., 1998; Hacke et al., 2010) is a key chemokine that attracts cells, including T lymphocytes, eosinophil granulocytes, macrophages, mast cells, and monocytes, to inflammatory sites. In general, CCL2 expression is activated after viral infection and recruits Langerhans and DCs, respectively. However, co-expression of the E6 and E7 proteins inhibits CCL2 expression in primary epithelial cells of the female genital tract (Hacke et al., 2010). An *in vitro* study (Nakamura et al., 1995) has suggested that HPV E7 interacts with interferon regulatory factor 1 (IRF-1), a significant regulator of cellular immune responses, to suppress CCL2 expression. This partly explains the almost undetectable activation of potent anti-tumor immune responses in primary HPV-infected epithelial tissues. It has been reported that humans lack a robust inflammatory response against primary HPV infection and progressively lose immune cells in the cervical stroma during CIN (Kleine-Lowinski et al., 2003; Hacke et al., 2010; Garrido et al., 2021). CIN is strongly associated with CCL2 expression in the tumor microenvironment. Habbous et al. (2012) suggested that as CIN progresses, levels of many hosts immune response markers, including the chemokine CCL2, are reduced. However, the specific mechanisms involved remain unclear. As epithelial dysplasia progresses to cervical cancer, macrophages, T cells, and LCs of the cervical mucosa are depleted. Restoring CCL2 expression can specifically improve the anti-tumor immunity of the host and block disease progression. Therefore, exploring the selective loss of CCL2 expression after E6/E7 transduction is crucial to blocking disease progression. However, in cell adhesion, the role of chemokines still needs to be explored in many ways. CXCL10 (Halle et al., 2017; Takeuchi and Saito, 2017) is associated with increased quantities of tumor-infiltrating CD8^+^ T cells, inducing the release of cytotoxic molecules (such as granzyme B and perforin) and apoptosis, which are associated with lower levels of cancer metastasis and improved patient survival in ovarian and colon cancer. CCL5 (Bruand et al., 2021) expression was found to be positively correlated with CD8^+^ T cell infiltration. T cell infiltration reportedly requires the secretion of CCL5 by cells in both epithelial cancer cell models and animal models. Zhang et al. (2020) suggested that CCL7 promotes anti-PD-1 therapy (CDC1) in a mouse model by recruiting conventional DCs to the tumor microenvironment to promote T cell proliferation. In addition, chemokines contribute to anti-tumor immunity by recruiting tumor-associated antigen-presenting cells, promoting their binding to T cells, and influencing tumorigenesis by interacting with tumor stem cell-like cells and stromal cells (Kombe Kombe et al., 2020). In summary, different lymphocyte subsets are recruited into the tumor microenvironment through different chemokine–chemokine receptor signaling pathways. The restricted expression of chemokines directly or indirectly leads to the failure of anti-tumor immunity.

Our study revealed 1,644 upregulated mRNAs and 1,261 downregulated mRNAs after E6/E7 overexpression. According to RNA-seq analysis, there is a tight connection between DEGs and cell adhesion, focal adhesion, leukocyte–endothelial migration, tight junctions, and Hippo and Wnt signaling pathways. KEGG analysis revealed that these DEGs were highly focused in cancer proliferation-related pathways, including the MAPK, Hippo, Wnt, and cell adhesion signaling pathways. It has been suggested that E6/E7 might exhibit crosstalk with these signaling pathways, affecting cell adhesion and limiting the immune response. CXCR4 (Zou et al., 1998; Goïta and Guenot, 2022) is a chemokine G protein-coupled transmembrane receptor released by stromal cells. Stromal cell-derived factor-1 (SDF-1; CXCL12), a ligand of CXCR4, induces B-cell proliferation and T-cell recruitment by activating CXCR4-expressing cells. Studies have demonstrated that the immune infiltration of B cells in the tumor microenvironment is associated with a survival advantage in patients with cervical (Abdul Rahman et al., 2020), breast (Hassel et al., 2021), and high-grade serous ovarian (Montfort et al., 2017) cancer; B cells are recruited through the binding of CXCL12 and CXCR4 (Greenbaum et al., 2013). Furthermore, CXCL12/CXCR4 enhances Src phosphorylation by activating MAPK or mitogen-activated protein kinase (MEK)/extracellular signal-regulated kinase (ERK) kinases (Kasina and Macoska, 2012). CXCR4-induced signaling cascades are commonly referred to as the “CXCR4–SDF-1 axis” and are frequently associated with lymphocyte trafficking and homing. To participate in the immune response (Peled et al., 1999), CXCL12 binds to CXCR4 to stimulate the firm adhesion of leukocytes by increasing integrin stability, which binds to intracellular adhesion molecules (ICAM-1) and vascular CAMs (VCAM-1). Researchers (Zheng et al., 2017) have used the CRISPR-Cas9 system in a cellular model to knock out CXCR4 and observed that colorectal cancer cell lines showed reduced adhesion to the ECM and endothelium, further demonstrating that CXCR4 is closely related to the Akt and type 1 insulin-like growth factor receptor (IGF1R) signaling pathways. In mouse models (Aggarwal et al., 2019), the CXCL12/CXCR4 axis enhances the function of M1-type macrophages, stimulates the production of inflammatory cytokines, and increases the phagocytosis of pathogens. Furthermore, it has been shown that CXCL12 primes IL-1 production in M2 macrophages by inducing its receptor CXCR4 and directly mediating the activation of CD4^+^T cells (Yu et al., 2020). Macrophages, an essential component of the innate immune system, are highly heterogeneous and malleable, and the reduced expression of CXCR4 might affect the dysregulation of M1/M2 phenotypes (Beneduce et al., 2019). This could play a vital role in the immune escape mediated by E6 and E7. These results suggest that CXCL12/CXCR4 is a potential therapeutic target for treating autoimmune diseases, including human immunodeficiency virus infection, cancer, warts, and immunodeficiency (Comba et al., 2020).

The expression of E6 and E7 resulted in the detection of many DEGs using RNA-seq and RT-qPCR. Fourteen of these hub genes were linked to cell adhesion (Chute, 2006; Teicher and Fricker, 2010; Paolillo and Schinelli, 2019; Marotta et al., 2021), leukocyte–endothelial migration (Yakovlev et al., 2019; Morein et al., 2020; Yan et al., 2021), stem cell signaling (Davidson et al., 2007; Bijlmakers, 2009), and epithelial–mesenchymal transition (Pastushenko et al., 2021; Wang et al., 2021). Based on these experimental and bioinformatics analyses, we hypothesized that HPV16 E6 and E7 signaling causes differential mRNA expression through direct or indirect mRNA pathways that regulate cell adhesion and chemokine secretion.

In this study, we used RNA-seq, RT-qPCR, and bioinformatics to validate the HPV-associated signaling pathways and possible downstream signaling molecules of the E6 and E7 proteins in human keratinocytes. This study provides a new direction and basis for further elucidation of the mechanism of E6/E7-mediated cell adhesion suppression. However, on the one hand, our study was restricted to *in vitro* conditions and the mode of interaction between CCL2 and hub genes is unclear in keratinocytes immortalized with both HPV E6 and E7 genes. The detailed mechanism will be investigated profoundly in our future studies. On the other hand, although some studies have indicated that FGF stimulation produces phosphorylation of E-cadherin and β-catenin on tyrosine residues, as well as increased E-cadherin localization to the cytoplasmic membrane and association with FGFR1 demonstrable by co-immunoprecipitation in the human pancreatic adenocarcinoma cell lines (BxPc3, T3M4 and HPAF), more direct interactions between hub genes need to be verified by co-immunoprecipitation or immunoblastic hybridization in our cellular models. The levels of mRNA only respond to the transcriptional aspect, while the process of post-translational modifications is highly variable, with different modifications resulting in different protein expression. Such differences may be influenced by post-transcriptional mechanisms such as methylation and acetylation. A previous study revealed that the level of m6A-modified mRNA is increased in cancer cells during the epithelial-mesenchymal transition of hepatocellular carcinoma cell metastasis (Lin et al., 2019). Although several studies have confirmed that the mRNA levels of certain hub genes such as CHD1, CDH2 (Castillo et al., 2016; Wang et al., 2020), IL-1β, EGF (Fu et al., 2020), BDNF (McDole et al., 2015), SOX2 (Scapin et al., 2019), and TLR4 (Jin et al., 2017) were consistent with the protein levels, it has not been validated in HaCaT-HPV16E6E7 cells. Considering this special feature, we will focus on discovering and exploring the differences in hub gene at the mRNA and protein levels in our future studies. It is undeniable that lentiviral vectors have an effect on gene expression, and we chose parental HaCaT as a negative control in our study, which makes it difficult to exclude the differential gene changes that would result from null loading at this time.

## Data availability statement

The data presented in this study are deposited in the SRA repository, accession number: PRJNA850539 can be found in the article/Supplementary material.

## Author contributions

RD and QR conceived and designed the experiments and revised the manuscript. RD and XL performed the experiments. RT carried out RNA-seq. RD, RT, SZ, TS, and QR analyzed the data. RD wrote the manuscript. All authors have read and approved the final manuscript.

## Footnotes

1
https://www.ncbi.nlm.nih.gov/bioproject/PRJNA850539

2
http://www.geneontology.org/

3
http://www.genome.jp/kegg/

4
http://www.broadinstitute.org/gsea

5
https://cn.string-db.org/

## Supplementary material

Supplementary Figure 1 Verification of HPV 16 E6/E7 Stable Expression HaCaT Cell Line Clone#4, #6, #10, #11 The results suggested that HPV 16 E6/E7 Stable Expression HaCaT Cell Line Clone#4, #6, #10, #11 all displayed significant overexpression of HPV16 E6 and E7. While, Clone#11 showed the highest expression of target gene, validated by RT-qPCR method.

Supplementary Figure 2 The original western blots of HPV16E6 with protein markers WB results showed that the expression of β-actin as an internal reference HPV16 E6 was reduced in protein level.

Supplementary Figure 3 The original western blots of HPV16E7 with protein markers WB results showed that the expression of β-actin as an internal reference HPV16 E7 was reduced in protein level.

Supplementary Figure 4 **(A)** RT-qPCR results that used β-ACTIN as internal control genes. **(B)** RT-qPCR results that used GAPDH as internal control genes. Verification of 14 hub gene RT-qPCR results were quantified by real-time PCR using GAPDH and β-ACTIN as internal control genes, respectively. It can be seen in bar graph that the verification is consistent with the results of Figure 1. ^***^*p* < 0.001, ^****^*p* < 0.0001.

Supplementary Figure 5 GESA analyses The vertical coordinate is the ES value and the horizontal coordinate is the sequencing of the gene. According to the result of gene sequencing, the genes in that pathway/term are scored plus and the genes not in that pathway/term are scored minus. The final maximum value obtained is the maximum value for that pathway/term. The points that are added will be marked with “vertical lines” below the graph.

Supplementary Figure 6 Verification of 14 hub gene Relative expression results of 14 hub gene were quantified by real-time PCR using GAPDH and β-ACTIN as internal control genes, respectively and shown by the bar graph whose horizontal and vertical coordinates represent the gene, names and relative expressions, respectively. The verification is consistent with the results of Figure 7. **p* < 0.05, ^**^*p* < 0.01, ^***^*p* < 0.001, and ^****^*p* < 0.0001.

Supplementary Material The differential expression analysis results (DESeq2_analysis_results) used in this study were uploaded, and these tables were placed in the Supplementary Material.
