# Supplementary figures and images for: Expression profiling of mRNA and functional network analyses of genes regulated by human papilloma virus E6 and E7 proteins in HaCaT cells
Source: Front Microbiol. 2022 Sep 14;13:979087. doi: 10.3389/fmicb.2022.979087 (PMC9515614; doi:10.3389/fmicb.2022.979087)

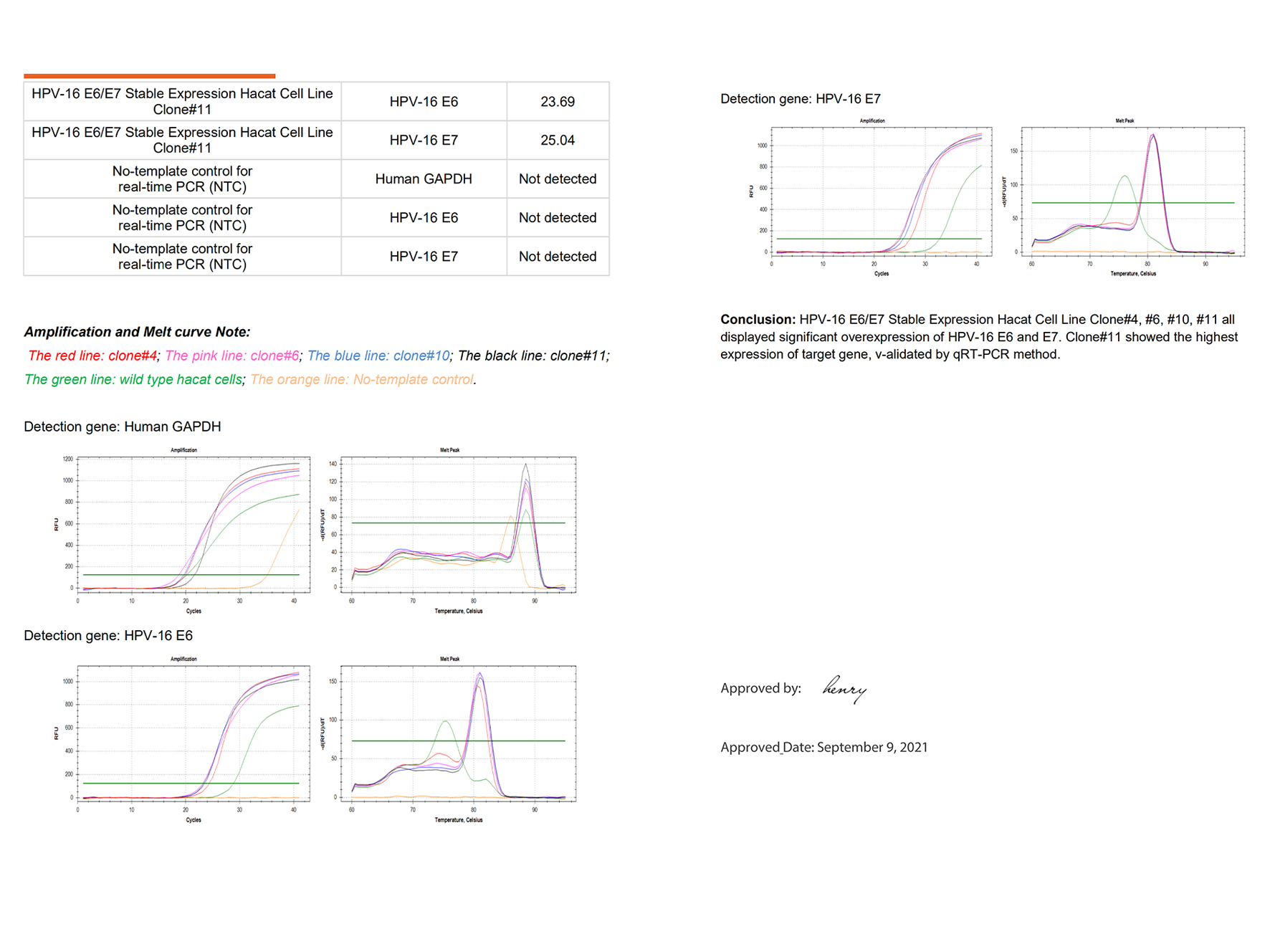

Supplement: Supplementary Figure 1 — Verification of HPV 16 E6/E7 Stable Expression HaCaT Cell Line Clone#4, #6, #10, #11 The results suggested that HPV 16 E6/E7 Stable Expression HaCaT Cell Line Clone#4, #6, #10, #11 all displayed significant overexpression of HPV16 E6 and E7. While, Clone#11 showed the highest expression of target gene, validated by RT-qPCR method. [file Image_1.TIF]

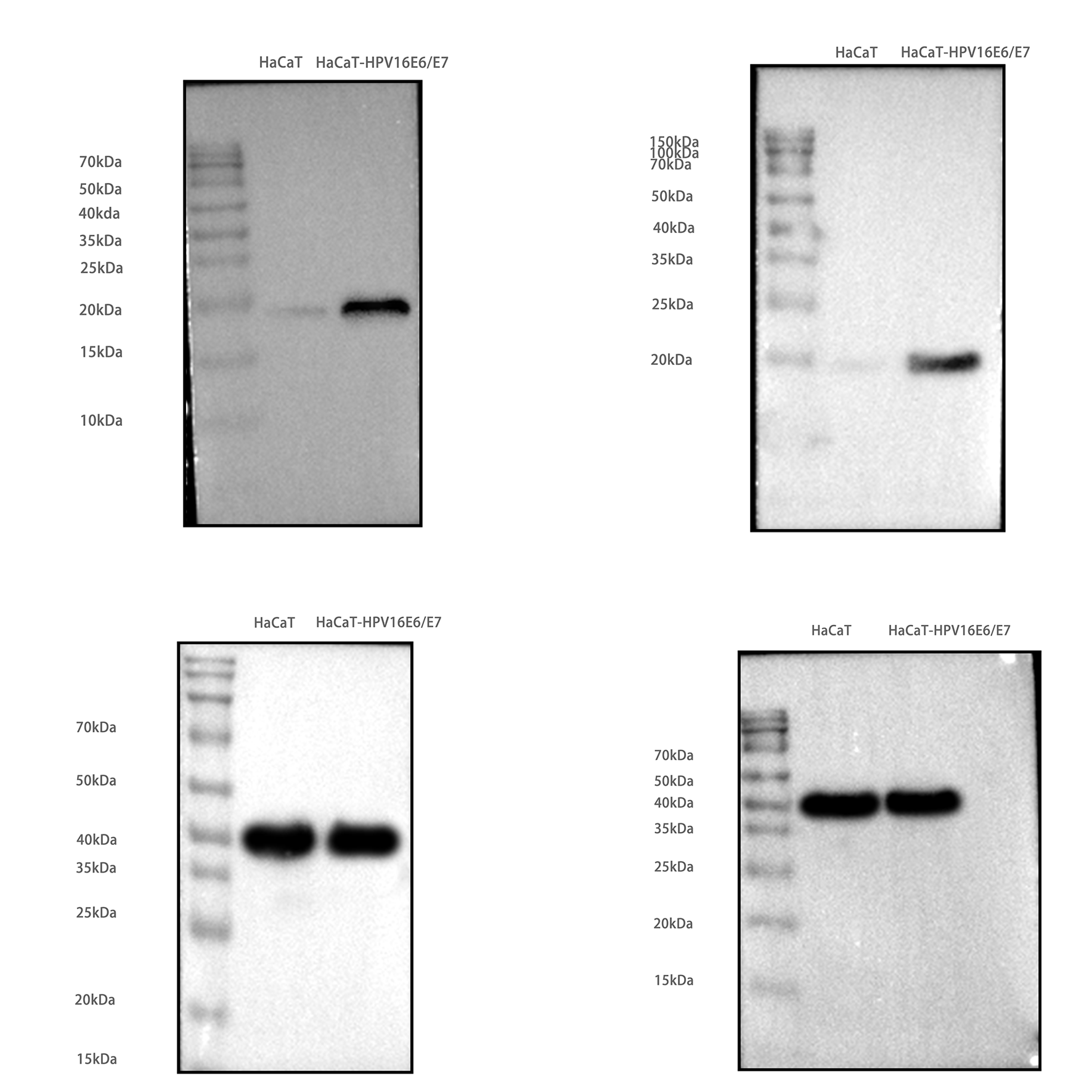

Supplement: Supplementary Figure 2 — The original western blots of HPV16E6 with protein markers WB results showed that the expression of β-actin as an internal reference HPV16 E6 was reduced in protein level. [file Image_2.TIF]

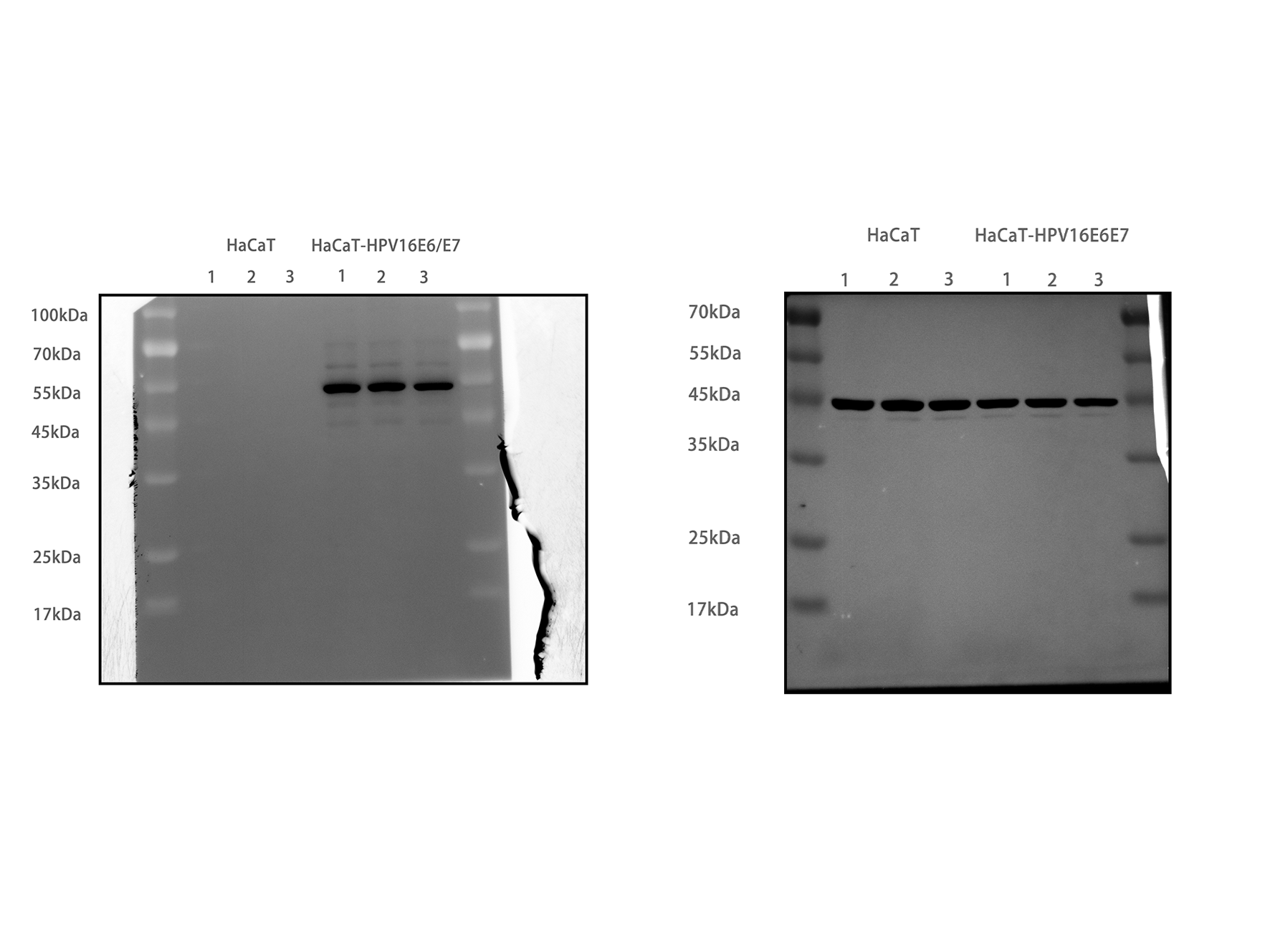

Supplement: Supplementary Figure 3 — The original western blots of HPV16E7 with protein markers WB results showed that the expression of β-actin as an internal reference HPV16 E7 was reduced in protein level. [file Image_3.TIF]

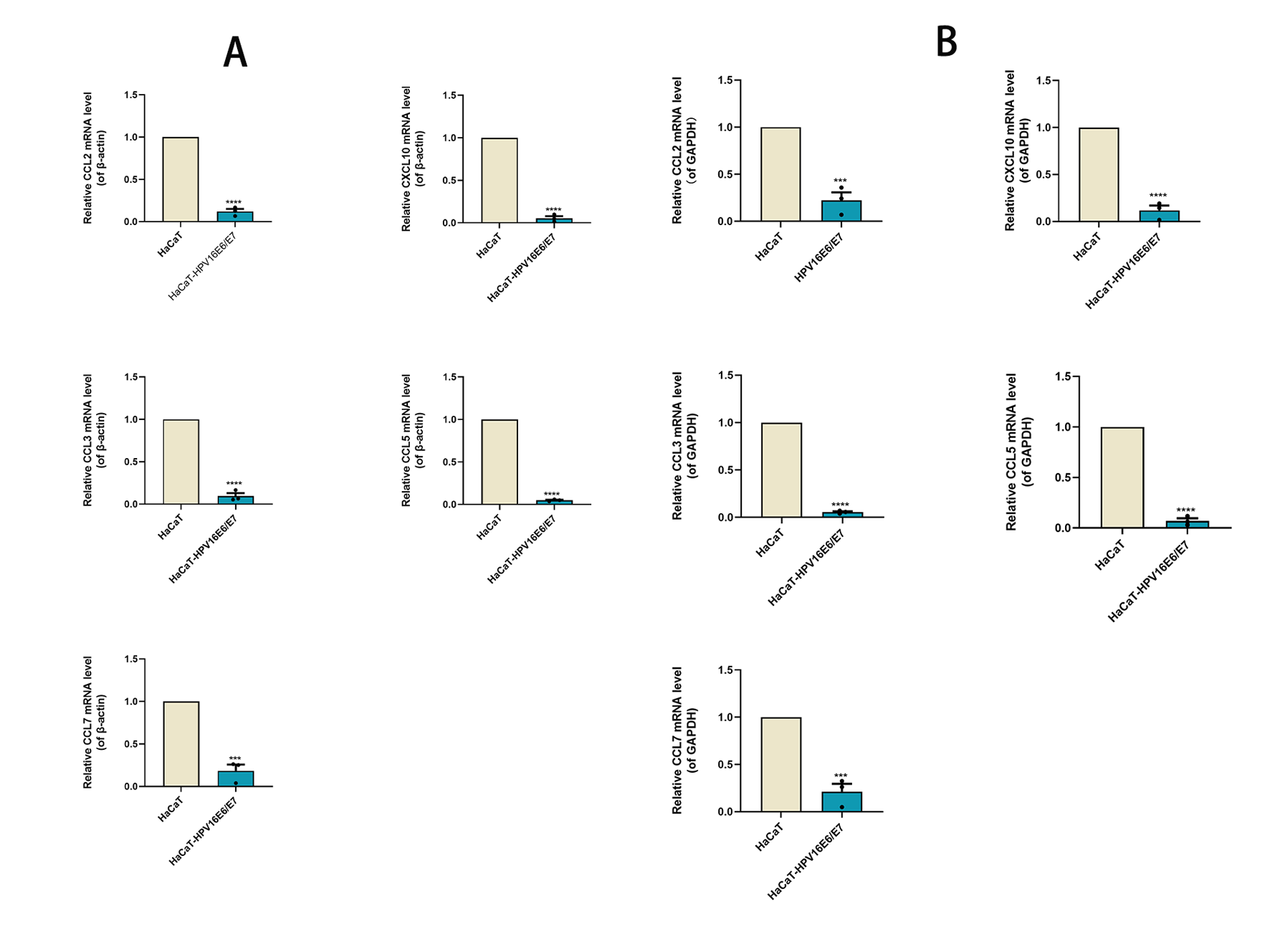

Supplement: Supplementary Figure 4 — (A) RT-qPCR results that used β-ACTIN as internal control genes. (B) RT-qPCR results that used GAPDH as internal control genes. Verification of 14 hub gene RT-qPCR results were quantified by real-time PCR using GAPDH and β-ACTIN as internal control genes, respectively. It can be seen in bar graph that the verification is consistent with the results of Figure 1. ***p < 0.001, ****p < 0.0001. [file Image_4.TIF]

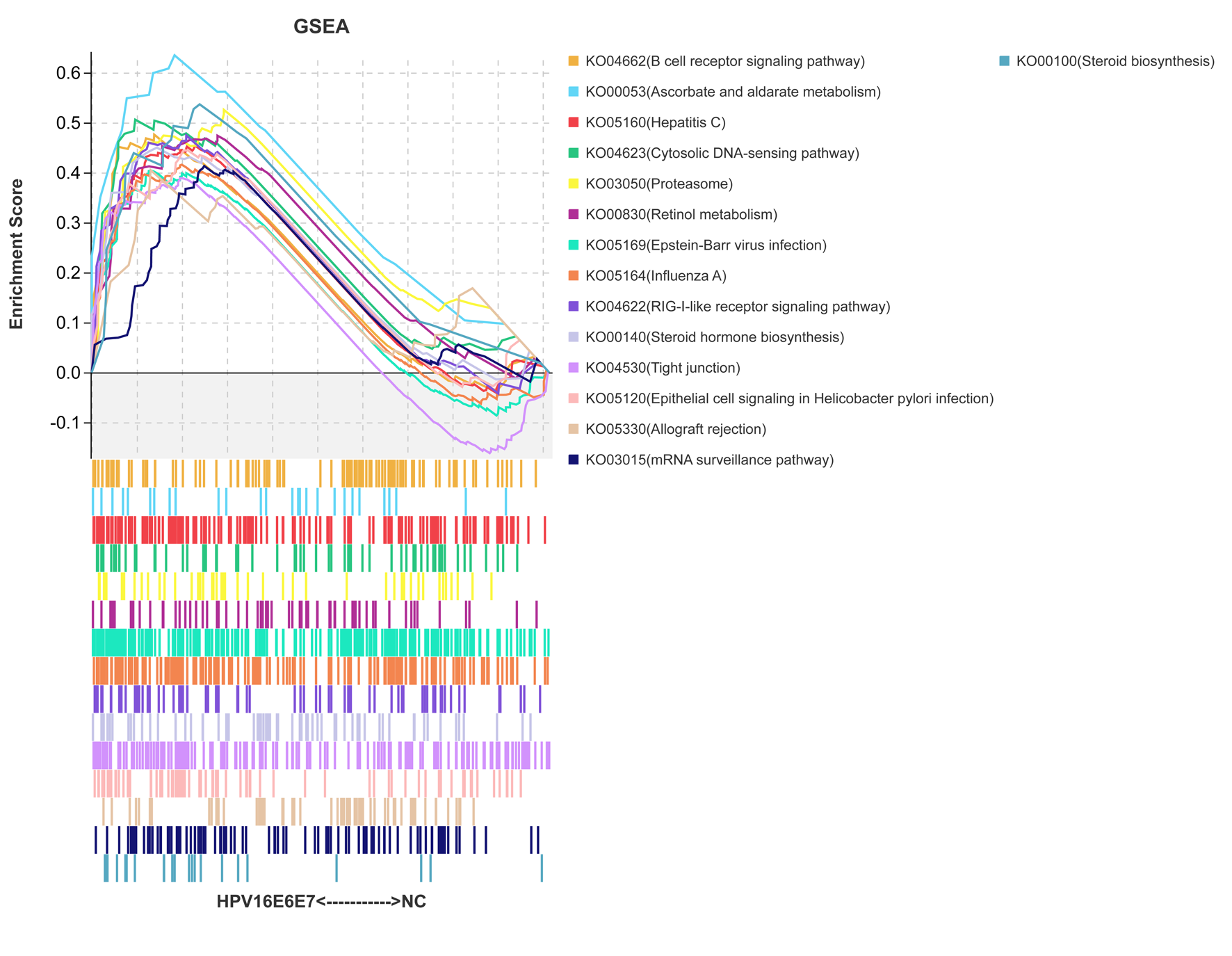

Supplement: Supplementary Figure 5 — GESA analyses The vertical coordinate is the ES value and the horizontal coordinate is the sequencing of the gene. According to the result of gene sequencing, the genes in that pathway/term are scored plus and the genes not in that pathway/term are scored minus. The final maximum value obtained is the maximum value for that pathway/term. The points that are added will be marked with “vertical lines” below the graph. [file Image_5.TIF]

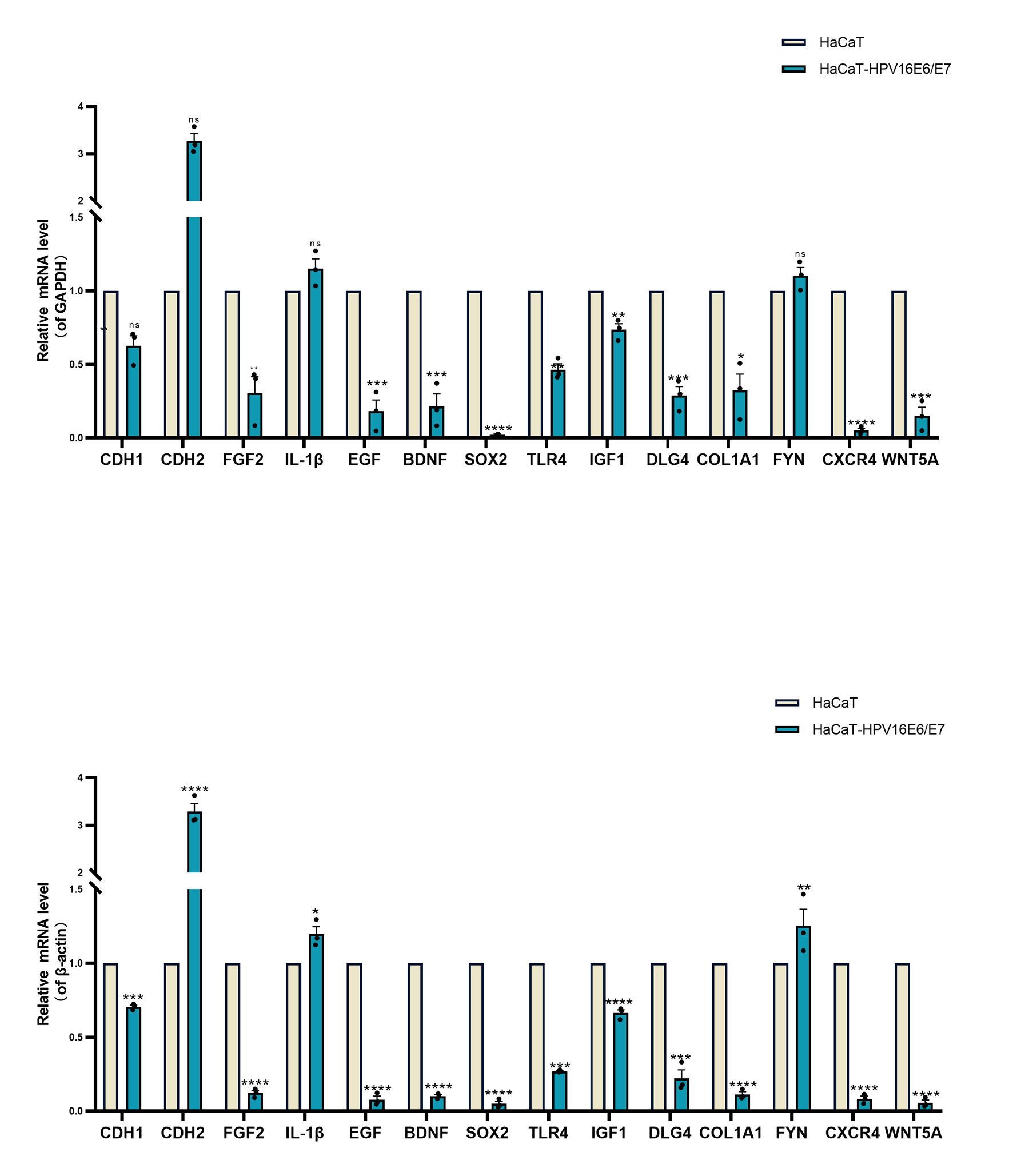

Supplement: Supplementary Figure 6 — Verification of 14 hub gene Relative expression results of 14 hub gene were quantified by real-time PCR using GAPDH and β-ACTIN as internal control genes, respectively and shown by the bar graph whose horizontal and vertical coordinates represent the gene, names and relative expressions, respectively. The verification is consistent with the results of Figure 7. *p < 0.05, **p < 0.01, ***p < 0.001, and ****p < 0.0001. [file Image_6.TIF]
